# Decoding the etiology of immune-mediated inflammatory diseases statistically

**DOI:** 10.3389/fimmu.2025.1610662

**Published:** 2025-06-17

**Authors:** Hesham ElAbd, Aya K. H. Mahdy

**Affiliations:** ^1^ Institute of Clinical Molecular Biology, Kiel University and University Hospital Schleswig-Holstein, Kiel, Germany; ^2^ Institute for Digestive Research, Lithuanian University of Health Sciences, Kaunas, Lithuania

**Keywords:** immune-mediated inflammatory diseases, immune repertoire, T-cell repertoire profiling, T-cell therapies, statistical analyses, etiology, immunogenetics

## Abstract

Immune-mediated inflammatory diseases (IMIDs) are incurable pathologies with an increased prevalence. Whereas different risk factors for IMIDs have been identified, such as microbial dysbiosis, diet, Epstein-Barr virus infection, the exact cause of most of these diseases remains unknown and it is thought to be a combination of environmental exposures and genetic predispositions. Despite their different clinical presentation, most IMIDs are genetically associated with variants at multiple immune-related genes, predominately with different human leukocyte antigen (HLA) alleles suggesting a strong pathological involvement of adaptive immune responses. However, antigens causing these diseases remain, in most cases, unknown. Using statistical analyses of the immune repertoire, several markers of antigenic exposures have been associated with IMIDs. Here, we discuss different approaches to identify disease-associated antigenic exposure markers and formulate a framework to test their causal role in IMIDs. We then discuss the potential contribution of risk HLA alleles to diseases development and lastly, we discuss how either antigens causing IMIDs or their signatures on the immune repertoire can be exploited therapeutically.

## There is an urgent need for an etiological understanding of immune-mediated inflammatory diseases

IMIDs are a group of pathologies where chronic inflammation is evidenced, leading to tissue destruction, remodeling and eventually a loss-of-function. These diseases can be organ-specific, such as multiple sclerosis (MS), which affects the central nervous system, or systematic, impacting multiple organs simultaneously such as systemic sclerosis (SC). Several risk factors have been implicated in the pathogenesis of IMIDs such as Epstein-Barr virus (EBV) (1), for example, epidemiological and molecular studies established a strong link between infectious mononucleosis and inflammatory bowel disease (IBD) (2, 3). Furthermore, dysregulated immune responses toward EBV have been observed in other IMIDs as well, *e.g*. MS (4–6), rheumatoid arthritis (RA) (7), systemic lupus erythematosus (8), and Sjögren’s syndrome (9). Whereas a mechanistic understanding of the pathological role of EBV in these diseases remains to be identified, several mechanisms have been proposed, such as molecular mimicry between EBV and human proteins, for example, EBNA1 and GlialCAM (10) and EBNA1 and C1q (11). Beside EBV, other disease-specific alterations have also been identified, such as increased antibody responses toward citrullinated peptides in RA (12) and an expansion of a specific group of unconventional T cells in Crohn’s disease (CD) (13, 14), which is a subset of IBD, among other disease-specific immune dysregulations.

As an exact cause for most of these diseases remains to be identified, treatments are mainly directed at inhibiting the inflammation, to clinically control disease symptoms and induce remission. This, arguably, partially non-specific inhibition of the immune system is achieved using different ways, such as anti-TNFs, anti-integrins and anti-cytokines antibodies, among others. Nonetheless, these therapies fail to introduce remission in all affected individuals, *i.e.* primary non-responders (15–17). Even primary responders might develop resistance to these therapies, *i.e.* secondary loss of response (15, 17), reaching what can be called a “therapeutic celling” at least in some diseases such as IBD (18). This problem is also aggravated by the lack of any approved prognostic marker for therapy response, despite ongoing efforts (19).

The prevalence of some of these IMIDs have increased significantly over the second half of the twentieth century, for example, the prevalence of IBD in the Olmsted County in the US, increased from 0.12% in 1960 to 0.63% in 2019 (20). Based on current estimates, it is projected that the prevalence of IBD will be ~1% in Canada in the upcoming decade (20–22). Besides IBD, other IMIDs are also prevalent in the population, for example, in the US alone there is between 400,000 (23) to 700,000 (24) individuals with MS and between 2 to 2.8 million individuals are living with the disease globally (25, 26). A higher prevalence is seen with RA with 17.6 million people affected globally (27) and with atopic dermatitis (AD) where more than 200 million individuals are living with the disease worldwide (28).

The combination of high prevalence, lack of accurate prognostic markers and the high cost of these medications is having a deleterious impact on the quality-of-life of affected individuals and healthcare systems. Thus, there is an urgent need for a better understanding of these diseases which could lead to more personalized therapies that induce a long-lasting remission in most patients as well as the development of preventive strategies, *e.g.* vaccines, in high-risk individuals.

## Disease-associated genetic variants do not predetermine the development of IMIDs

With the rise of genome-wide association studies (GWAS) during the last couple of decades, the genetic signatures of IMIDs have been heavily investigated (29–34). For example, using the genetic data of 47,429 individuals with MS and 68,374 controls, more than 233 variants were associated with MS, 32 of them were located within the extend HLA loci (35). Similarly for RA, a recent meta-analysis spanning 35,871 individuals with RA and 240,149 controls from different ancestries identified 124 loci associated with RA (32). Similar meta-analyses were conducted in other IMIDs such as psoriasis, where a recent study has identified 109 loci that are implicated in the disease using a large cohort of 36,466 cases and 458,078 controls (36). Also, in AD 71 genetic variants were implicated in the disease by analyzing the genetic background of 65,107 individuals with AD and 1,021,287 controls (33).

A common denominator among these IMIDs was the lack of clear causative genetic mutations, as opposed to Mendelian genetic diseases, instead there was multiple associations to different common genetic variants. For most IMIDs, these associations resolved to genetic variants in different innate and adaptive immunity-related genes and loci (32, 33, 35, 36) such as the human leukocyte antigen (HLA) loci. Most of the associated HLA alleles have a moderate association odds ratio (OR) and were frequent in the study population in general. For example, in MS, the strongest genetic association is with the HLA-DRB1*15:01 with an OR of ~3 (37–39). Whereas the frequency of this alleles varies across populations and ancestries, it is frequent in European populations (frequency >10%) (39).

This implies that millions of individuals are carrying disease-associated HLA alleles and are not affected, at least symptomatically, with these diseases. This is clearly seen in celiac disease (CeD) which is strongly associated with HLA-[DQ2.2, DQ2.5 and DQ8] alleles (40, 41), nonetheless, not all carriers of these alleles are developing CeD. Thus, other environmental factors besides genetics are contributing to IMIDs such as gluten in the context of CeD.

## The adaptive immune system records previous antigenic exposures as V(D)J generated sequences

HLA proteins are a central hub for communication among different parts of the immune system. They are classified into two classes, class I which presents peptides to CD8^+^ T cells and class II which presents peptides to CD4^+^ T cells. Thus, they convey critical information about potential peptide antigens between all nucleated cells and CD8^+^ T cells, between B cells and CD4^+^ T cells and between dendritic cells and T cells. A hallmark of adaptive immunity is the formation of an immunological memory after an antigenic exposure. This immune memory is composite of three main elements, first, a unique immune receptor that recognize different antigenic peptides from pathogens, *i.e.* T and B cell receptors, TCRs and BCRs, respectively. These receptors are generated via V(D)J recombination events and are engraved in the DNA encoding the TCR and the BCR of this antigen-specific T and B cells.

Nonetheless, before we continue our discussion, we need to highlight important distinctions between TCRs and BCRs, namely, somatic hypermutation and class-switching which are exclusive to BCRs. Somatic hypermutation is a process used by B cells to enhance the affinity of their receptors, *i.e.* BCRs, toward a specific antigen, through random mutations introduced in their immune receptor chains followed by selection of mutations that increase the affinity of the BCR toward its cognate antigen, via an interaction with follicular helper T (Tfh) cells (42). This results in a family of related BCRs that bind the antigen with varying affinities, that is, a phylogenetic tree of “evolutionary-related” sequences that respond to the initial antigenic exposure (43). A second mechanism that is also specific to BCRs is class-switching where the isotype of the immunoglobin heavy chain is changing from μ, which is formed during the early phase of antigenic exposure, to other isotypes, for example, α, γ or ϵ which are used in IgA, IgG and IgE antibodies, respectively. Nonetheless, beside these differences between BCRs and TCRs, an antigenic exposure is associated with the formation of long-lived plasma cells and memory B cells that respond to this infection and record this exposure in the form of a DNA-encoded V(D)J recombination sequences (44).

**Figure 1:**
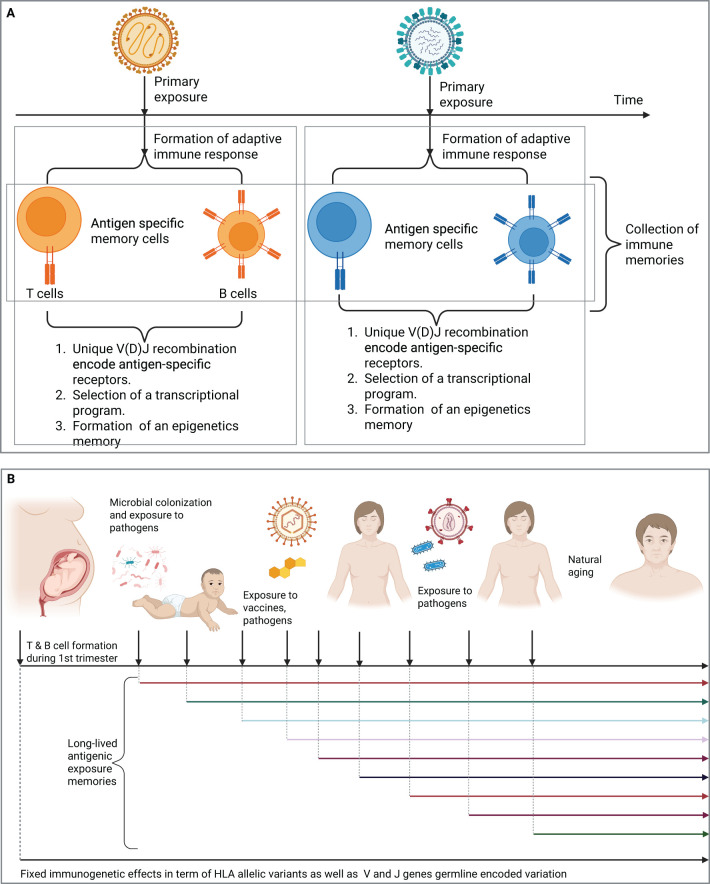
T and B cell repertoires record the antigenic exposure history of an individual. **(A)** The formation of a long-lived adaptive immune memories after the exposure to two distinct viruses, each of which will result in the formation of a distinct immune memory that records this antigenic exposure **(B)** The immune repertoire contains long-lived adaptive immune memories the records previous antigenic exposure histories. Created in BioRender. Elabd, H. (2025) https://BioRender.com/tn5de67.

The second part of an immune memory is a transcriptional program that shapes the behaviors of antigen-specific T and B cells and govern the phenotype of these cells, for example, T helper 1, 2, or 17. Also, naive B cells can follow different developmental trajectories after an antigenic encounter, for example, they can develop into short-lived plasma cells, into germinal center (GC) B cells or into GC independent memory B cells (44). The last part is an epigenetic memory, which enforces and constrains the formed transcriptional program of these antigen-specific T and B memory cells (**Figure 1A**). These formed immune memories are mostly long-lived and provide protection against repeated infections by the same pathogen.

Hence, as we age, we accumulate more antigenic exposures, either from natural infections, or vaccines, each of these exposures elicit the formation of an immune memory, resulting in the accumulation of memory cells that record this exposure history. Before we continue our discussion, we need to introduce two temporal events, first, a starting point, which will be the 1^st^ trimester of gestation in humans where T and B cells begin to form (**Figure 1B**). Indeed, different compartments of the adaptive immune system develop at different stages of gestation for example, thymic development of T cells beings in the 1^st^ trimester, however, T cells egress from the thymus at the beginning of the 2^nd^ trimester, between the 12^th^ and 14^th^ week of gestation (45–47).

Second, a sampling timepoint, it is the timepoint of sampling a subset of the immune repertoire (**Figure 1B**). Based on these two events, the immune repertoire is defined here as the collection of immune memories, *i.e.* exposure histories, accumulated between the beginning and the sampling timepoint. For the sake of simplification, we are going to narrow down the definition of immune memories into the unique collection of V(D)J generated sequences. It is also worth mentioning that beside memory cells, the repertoire also contains V(D)J sequences from naive cells, which have not encountered their cognate antigen yet. Additionally, it contains V(D)J recombination sequences derived from effector cells responding to ongoing infections. For the sake of clarity, we focus on the memory compartment of the immune repertoire unless stated otherwise.

A powerful method to study the collection of V(D)J recombination events encoding immunological exposure histories, is bulk immune receptor sequencing (**Supplementary Figure S1A**) (48). Nonetheless, it has three main limitations, first, it only provides the sequence of the generated receptor and not the antigen to which it binds. Second, the temporal order or exposure histories is almost not-captured, unless it is a very recent or an ongoing exposure that results in the expansion of some V(D)J recombination sequences (**Supplementary Figure S1B**). Third, it does not reveal the functional state of cells expressing these receptors, for example, Th1, Th2, Th17, among others. Furthermore, bulk immune-sequencing methods do not provide the full sequence of the immune receptor only part of it, for example, in case of TCRs, only the alpha (TRA) or the beta (TRB) chain, that is, the pairing information is lost in bulk repertoire profiling experiments.

Whereas the pairing information of TCRs and the transcriptional landscape of cells expressing these receptors can be identified via single-cell T cell receptor sequencing either using short-reads (49) or long-read sequencing (50), this method has several limitations. First, it is expensive, labor intensive and provides a shallow profiling of the repertoire where only few 1000s of clonotypes are profiled using single cell approaches, while in bulk immune sequencing 100,000s of clonotypes can be identified (48). Second, it requires access to intact cells, *e.g.* fresh or frozen PBMCs, which possess logistical problem when profiling the repertoire of thousands of samples. Lastly, if the aim is to generate pairing information without information about the cell type, then probabilistic mapping of profiled immune receptor chains using frameworks, such as pairSEQ (51) and TIRTL-Seq (52) might be a more cost-efficient approach. Hence, by integrating these different frameworks and methods, a better understanding of different aspects of immune receptor chains can be obtained, for example, using bulk TCR-Seq to profile the repertoire of thousands of individuals. Then, utilize probabilistic pairing to obtain the pairing information for candidate clonotypes across hundreds of individuals and lastly, using single cell technologies to understand the transcriptional landscape of these individuals in tens of samples.

## Genetics has a fixed, robust effect on the formed immune repertoire that can be studied statistically

Before we delve deeper into how immune repertoires can be investigated to identify the etiological causes of IMIDs, we need to distinguish between two factors shaping the repertoire. First, fixed effects that is genetically predetermined regardless of antigenic exposures and second dynamic effects that result from a combination of genetic predeterminants and antigenic exposures. The fixed effects have three pillars, (i) germline encoded variation in the V and J genes, which forms the basis for V(D)J generated sequences (53–55). Second allelic variation in the HLA region (56–58) and third variation in other genomic loci (59). HLA proteins, regardless of any antigenic exposure, have a major impact on shaping the formed T cell immune repertoire, because of thymic selection. Different HLA proteins present different peptides to T cells, as shown previously by others and us (60–63), and during positive selection only V(D)J recombination sequences able to recognize self-peptides loaded into HLA proteins receive survival signal. Other genetic variants can have an influence by biasing the process of V(D)J recombination prior (59) to selection either by HLA proteins or by having coding variants that upon presentation by HLA alleles will shape T-cell selection. Alternatively, other somatic genes might encode for signaling molecules that change the perception and the execution of T and B cells to an antigenic stimulus (64, 65).

From a molecular perspective, HLA exerts two effects on the immune repertoire, first, it biases the frequency of utilizing different V genes in the repertoire (57). Additionally, HLA proteins have a strong effect on the frequency of amino acids in the complementarity-determining region 3 (CDR3) (56). Thus, prior to any antigenic exposure, an interaction between the germline encoded genes, HLA allelic variants and other coding and non-coding variants will shape the formation of naive T cells primarily by shaping which V(D)J recombination is selected. Thus, forming the base to which immune memories will be formed upon antigenic exposures. Additionally, the generated TCRs can also shape the transcriptional landscape of the generated naive cells, for example, different TCR signaling intensities can module the differentiation of double-positive T cells into either CD8^+^ or CD4^+^ single-positive T cells (66). These sequence features can also play a role in the fate-determination process of regulatory T cells (67). Hence, an interaction among these different factors will have a strong effect on shaping the generated repertoire not only in terms of sequence diversity but also the functional landscape.

Using large-scale statistical analyses of the immune repertoire, the fixed effect of HLA proteins on the immune repertoire can be elucidated. For example, using >5,500 paired T cell immune repertoire and HLA genotypes, we were able to discover hundreds of thousands of clonotypes associated with tenths of HLA alleles (68). These clonotypes could accurately impute the carriership of these HLA alleles, indicating the strong impact of HLA protein on shaping the generated immune memories. Using different statistical frameworks, the impact of variable HLA sites on the frequency of amino acids in the CDR3 of the TCR beta chains (69) was studied by others (56) and us (69). Highlighting several paths by which HLA proteins exhibit an effect on the formed immune repertoires.

## Identifying disease-associated shared antigenic exposures markers statistically

An immune memory is formed upon an antigenic exposure that results in the induction of a memory cell, the formation of these memory cells depends on the antigen, and fixed effects encoded genetically. With current technologies we can sequence V(D)J events, but unfortunately, we cannot, in most cases, decode their antigenic specificities, resulting in a trajectory of unknown antigenic exposures (**Supplementary Figure S1B**). Whereas newer technologies developed to decode the antigenic specificities of immune receptors, such as T-Scan (70), TScan-II (71) and receptor–antigen pairing by targeted retroviruses (RAPTR) (72), they require a rationally selected library of candidate TCRs, as well as peptide-HLA complexes. This represents a major hurdle for identifying the etiological causes of IMIDs for multiple reasons, first, in most cases neither the antigen nor the exact TCR(s) deriving these diseases are known. Second, the immune repertoire is extremely diverse and personal, that is, most clonotypes are observed in one individual and are not shared among individuals. Thus, it is not feasible neither financially nor logistically to conduct these assays on all T cells of a cohort of individuals living with an IMID of interest. Third, there might be a long variable time span between the causative antigenic exposure and disease development, which is evidenced in some IMIDs, *e.g.* MS (5) which complicates the identification process of antigens implicated in the disease. Hence, a narrowing down of candidate T cells involved in the disease is needed.

Assuming that there is a specific antigen or a group of antigens that are causing IMIDs, then within individuals having the same IMID and sharing the fixed-repertoire effects, *e.g.* similar HLA background, we expect the same immune memories toward this exposure to be formed. Thus, we expect some V(D)J recombination sequences to be shared among individuals with a specific IMIDs relative to individuals without this IMID. These shared V(D)J sequences represent the exposure signature of the disease, *e.g.* the memory T cells associated with an antigenic exposure implicated in the disease. From large repertoire profiling studies, it was observed that most of the immune repertoire is private, that is, most responses are detected in only one individual, and that shared or public immune responses represent a small fraction of the repertoire (73). These shared responses might represent an exposure marker toward prevalent antigenic exposures, for example, common viral and bacterial infections (74, 75). Our ability to identify shared clonotypes involved in responding to a known antigenic exposure by comparing the repertoires of exposed to non-exposed (73, 76, 77), provides evidence to suggest that an IMIDs-associated antigenic exposures can be identified by comparing the repertoire of cases and controls (78).

Nonetheless, relative to disentangling the antigenic exposure of a specific infectious agent, different factors might complicate our analysis to identify exact antigenic exposures implicated in IMIDs. First, the likelihood of an antigenic exposure causing the disease, which is similar to the concept of “penetrance” in genetics. Here, some antigenic exposures might not exhibit a perfect or near perfect penetrance, where having the exposure does not guarantee a certain likelihood to develop the disease, just an increase in the odds of developing the disease. A perfect example of this is EBV, which have been implicated in multiple IMIDs as discussed above, however, it is a very prevalent infection where more than 95% of the population is affected.

Second, some antigenic exposures might generate an immune response in almost everyone in the population. For example, in phage-immunoprecipitation (PhIP-Seq) (79, 80) studies of the immune repertoire, multiple antigens have been shown to be recognized by almost every individual in the population with a prevalence of the immune response that is >95% (81–83). While these antigens might be recognized by the immune system of every individual it does not imply that they will be recognized by the same immune receptor, or that the epitope is going to be mapped to the same part of the protein in every individual. Hence, differences between individuals with and without IMIDs, might not be *per se* in the antigenic exposure but in the antigenic region, or the exact epitope, targeted by the immune system.

As a result, to disentangle disease signatures in IMIDs, a methods that can identify immune signatures at the epitope level is needed. This method should be able to identify antigenic exposures at the infectious agent level, at the antigenic protein of this pathogen and lastly at the epitope level, that is sub-antigenic protein level. A powerful method to identify these disease signatures is to analyze the T cell repertoire of thousands of individuals statistically to identify disease signatures. Here, the immune repertoire of T cells, which recognize short class of peptides, between 9 and 17 amino acids, presented by either HLA-I or HLA-II proteins is analyzed which provides us with an epitope-based mapping of immune responses. By statistically investigating the T cell repertoire of >5,000 individuals with IBD and >5,000 healthy controls we were able to identify >1,800 distinct V(D)J recombination sequences implicated in IBD (84).

## Decoding the etiologies of IMIDs is similar to solving a temporal-credit assignment problem but with incomplete action history

**Figure 2:**
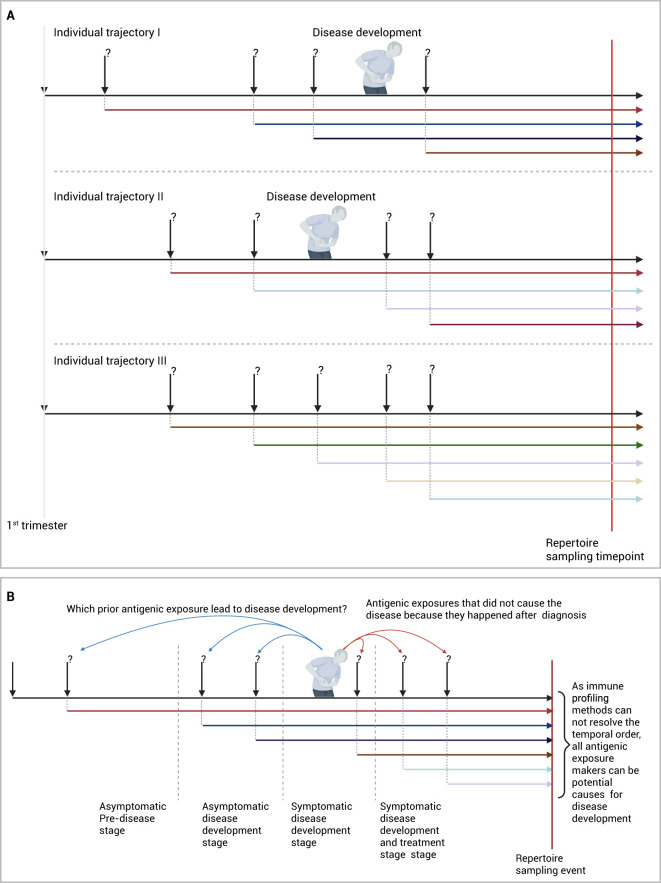
Identifying antigenic exposures deriving IMIDs. **(A)** Identifying diseases-associated antigenic exposures from cross-sectional studies. **(B)** An alternative framework to identify disease-causing antigenic exposures through longitudinal sampling of pre-clinical individuals. Created in BioRender. Elabd, H. (2025) https://BioRender.com/2cvfi4o.

While the approach described above provides an unparallel opportunity to identify disease etiologies, it has limitations primarily rated to the lack of temporal exposure order as most repertoire profiling methods provides a collection of immune memories without a temporal order. Given that most studies depend on a cross-sectional study design (**Figure 2A**) that includes individuals after their diagnosis with the disease, proving causation between the associated V(D)J sequences and the disease is not possible. Because these shared V(D)J sequences can be a consequence of the diseases instead of being the cause of the disease, *i.e.* a reverse-causation (**Figure 2B**).

A solution to this problem would require the temporal order of antigenic exposures before and after diagnosis to be resolved cohort-wide, to identify which antigenic exposure caused the disease and which exposure was caused by the disease. In essence, this will reduce the problem of identifying etiological factors into a temporal credit assignment problem, where exposures can be thought of as actions and developing the disease can be thought of as a reward. Hence, the solution to this problem becomes finding the action or series of actions, here antigenic exposures, responsible for disease development, that is, the reward in this formulation.

Nonetheless, resolving the temporal order of immune exposures from a profiled immune repertoire is still not possible with current technologies. A more practical approach will be to sample the repertoire of the study cohort across multiple timepoints ideally before disease development. This can be done using large-scale prospective or retrospective cohorts where multiple samples are collected from individuals before they develop the disease, as well as after they develop the disease (5, 85–87). By decoding the immune exposure across multiple points, a better understanding of exposures responsible for developing the disease can be obtained (**Figure 2B**). Given the high cost of immune profiling and the low incidence rate of most of these diseases, focusing the profiling on high-risk individuals, *e.g.* patients’ relatives (88), might provide a cost-efficient way to identify antigenic exposures causing the disease.

## Which or when, the importance of exposure timing

In our discussion so far, we have focused on identifying antigens deriving the disease regardless of the timing of exposure, which might be critical for shaping the outcome of the disease. For example, infectious mononucleosis, which is predominantly caused by an EBV infection has been implicated in many chronic inflammatory diseases, *e.g.* MS (5, 6, 89), RA (7, 90), and IBD (2, 91), among others. While EBV infection is commonly associated with infectious mononucleosis in adults, this rarely happens in children (92), suggesting that the same antigenic exposure can lead to different outcomes based on the timing of exposure. Beside biological age, previous exposures can also have a strong influence on shaping the outcomes of an exposure (93), hence, the exposure trajectory plays important roles in shaping the generated outcome.

## Do we need to identify etiological factors causing IMIDs to treat these diseases?

Given the high prevalence of IMIDs and their associated burden on health care systems, there is an urgent need for developing better therapeutic strategies to treat these diseases. However, do we need to know the etiology to effectively treat these diseases? By identifying V(D)J sequences associated with ankylosing spondylitis (78, 94) and depleting T cell populations containing these V(D)J sequences a novel therapy that induce remission in ankylosing spondylitis patients was developed (95). Whether this approach would generalize to other diseases with different etiologies and driver-antigens remains an open question.

## Can we escape the inevitable?

In some cases, antigens causing and deriving the disease are common environmental exposures, *e.g.* gluten in CeD, making avoiding the exposure a practical approach to control the disease (**Supplementary Figure S2A**). Nonetheless, these avoidance approaches still have challenges, for example, a gluten-free diet is expensive with a limited set of options available in the market (96) and the risk of gluten contamination and mislabeling exists (97). As a result, different pharmacological interventions are being developed to treat CeD, for example, peptidases to digest gluten before it can elicit an immune response and intestinal barrier regulators (98).

In other cases, disease-causing antigenic exposures might be a common viral infection, such as EBV which infects >90-95% of the population, making avoidance a much harder problem. While identifying antigenic exposures causing the disease might provide a promising strategy for therapeutic interventions, developing a preventive strategy might not be trivial, *e.g.* avoiding the exposure might not be possible. Alternatively, other sophisticated approaches, such as vaccines for either inducing tolerance (**Supplementary Figure S2B**) (99), or protection against a particular disease-causing exposure (100) might be needed to prevent disease development. Lastly, by identifying disease-causing antigens and their cognate immune cells, technologies like monoclonal antibodies targeting the V(D)J recombination of these cells (**Supplementary Figure S2C**) (95) and chimeric autoantibody receptor (CAAR) T cells can be used to specifically deplete immune cells driving the disease (**Supplementary Figure S2D**) (101).

## Concluding remarks

There is an urgent, unmet need for a better understanding of IMIDs. The strong association between most of these diseases and several HLA alleles suggest an import role for adaptive immunity, specifically, T cell mediated responses in the disease. Nonetheless, the antigens causing these diseases remain to be identified, here we discussed several approaches and frameworks to identify antigenic exposure markers implicated in the disease, as well as potential experimental designs to establish causation. Despite the recent progress, many open questions remain to be addressed, for example, can we develop a more sample-efficient algorithm to identify disease-associated clonotypes? Current approaches depend on utilizing large cohorts of cases and controls; however, it is a very costly approach and not suitable for rarer diseases where assembling large cohorts is not feasible. Additionally, can we infer the antigenic specificity of a given V(D)J recombination event computationally? Can we infer the antigenic exposure trajectories, *i.e.* the order of antigenic exposures from the profiled T cell repertoire? Further, what is the contribution of private immune responses to IMIDs, relative to shared or public responses? In conclusion, the identification of disease-causing antigens or even their corresponding V(D)J sequences, will have a transformative utility on the development of therapeutic and preventive strategies for IMIDs.

## Data Availability

The original contributions presented in the study are included in the article/**Supplementary Material**. Further inquiries can be directed to the corresponding authors.
