# Supplementary material for: Decoding the etiology of immune-mediated inflammatory diseases statistically
Source: Front Immunol. 2025 Jun 17;16:1610662. doi: 10.3389/fimmu.2025.1610662 (PMC12209366; doi:10.3389/fimmu.2025.1610662)
Supplement: Supplementary file 1 [file DataSheet1.docx]

Supplementary figures


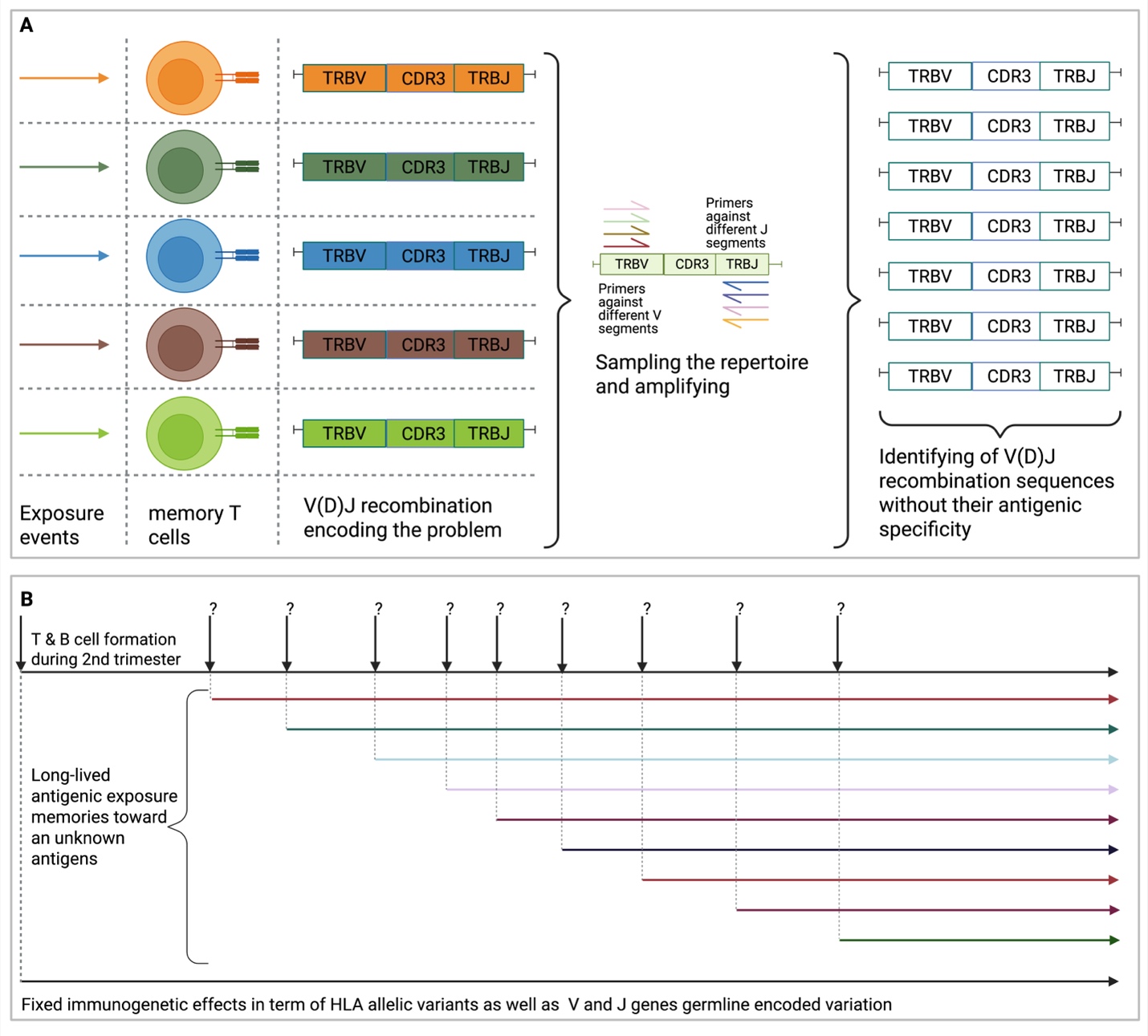


**Figure S1:** limitations of current immune profiling technologies. (**A**) technologies such as bulk repertoire sequencing provides the sequences of antigenic binding chains encoded by V(D)J recombination sequences representing the immune memory to different antigenic exposures. However, it does not provide information about the temporal order of antigenic exposures or about the antigenic specifies of these V(D)J sequences. (**B**) the immune repertoire from the perspective of bulk immune sequencing technologies, where the repertoire is made from a series of mostly unknown exposures at undefined intervals. Created in BioRender. Elabd, H. (2025) https://BioRender.com/q48qjtb


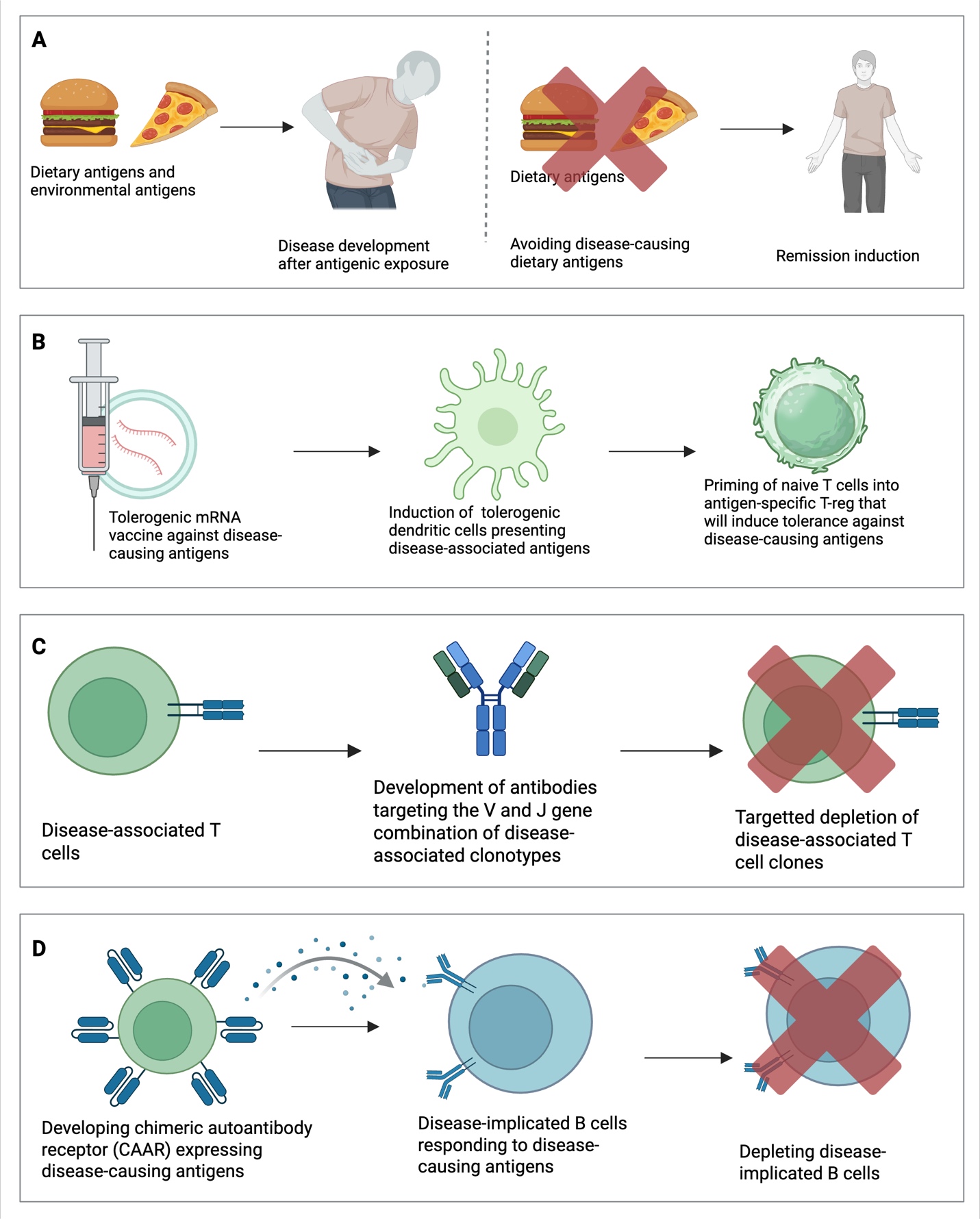


**Figure S2**: different therapeutic strategies to treat immune-mediated inflammatory diseases after identifying either the antigens involved in the disease and/or the exact immune cells driving the disease. (A) avoidance of disease-causing antigens might be a practical approach to control the disease. (**B**), reprogramming of immune responses toward disease-implicated antigens using tolerogenic mRNA vaccines. (**C**) depletion of disease-associated clonotypes using antibodies targeting specific V/J combinations implicated in the disease. (**D**) depletion of disease-implicating B cells using chimeric autoantibody receptor (CAAR) T cells expressing disease-implicated antigen on its surface. Created in BioRender. Elabd, H. (2025) <https://BioRender.com/5clexr0>
